# Exactin: A specific inhibitor of Factor X activation by extrinsic tenase complex from the venom of *Hemachatus haemachatus*

**DOI:** 10.1038/srep32036

**Published:** 2016-08-25

**Authors:** Vallerinteavide Mavelli Girish, R. Manjunatha Kini

**Affiliations:** 1 Department of Biological Sciences, Faculty of Science, National University of Singapore, Singapore 119260, Singapore; 2 Department of Biochemistry, Medical College of Virginia, Virginia Commonwealth University, Richmond, Virginia 23298, USA; 3 University of South Australia, School of Pharmacy and Medical Sciences, Adelaide, South Australia 5001, Australia

## Abstract

Unwanted clots lead to heart attack and stroke that result in a large number of deaths. Currently available anticoagulants have some drawbacks including their non-specific actions. Therefore novel anticoagulants that target specific steps in the coagulation pathway are being sought. Here we describe the identification and characterization of a novel anticoagulant protein from the venom of *Hemachatus haemachatus* (African Ringhals cobra) that specifically inhibits factor X (FX) activation by the extrinsic tenase complex (ETC) and thus named as exactin. Exactin belongs to the three-finger toxin (3FTx) family, with high sequence identity to neurotoxins and low identity to the well-characterized 3FTx anticoagulants-hemextin and naniproin. It is a mixed-type inhibitor of ETC with the kinetic constants, Ki’ and Ki determined as 30.62 ± 7.73 nM and 153.75 ± 17.96 nM, respectively. Exactin does not bind to the active site of factor VIIa and factor Xa based on its weak inhibition (IC_50_ ≫ 300 μM) to the amidolytic activities of these proteases. Exactin shows exquisite macromolecular specificity to FX activation as compared to factor IX activation by ETC. Exactin thus displays a distinct mechanism when compared to other anticoagulants targeting ETC, with its selective preference to ETC-FX [ES] complex.

Blood coagulation, a hemostatic response to vascular injuries, is a highly synchronized cascade that involves sequential activation of blood coagulation factors leading to the formation of fibrin clot^1^. Any imbalance in its regulation can lead to either unwanted clot (thrombosis) or excessive bleeding (hemorrhage)^2^. Vascular occlusion due to thrombosis in vital organs, as in cardiovascular and cerebrovascular diseases, results in high morbidity and mortality. Anticoagulants prevent the incidence of debilitation and death from unwanted clots^3^. An estimated 0.7% of the western population receives oral anticoagulation therapy with heparin and vitamin K antagonists^4^. The former mediates its anticoagulant activity by enhancing the inhibitory activity of antithrombin, while the latter exhibits their activity by interfering with the hepatic synthesis of vitamin K-dependent blood coagulation proteins^5^,^6^. However, these oral anticoagulants have several limitations. Heparin binds non-specifically to other plasma proteins and endothelial cells resulting in its reduced bioavailability and hence anticoagulant activity. In some individuals, it also interacts with platelet factor-4 resulting in heparin-induced thrombocytopenia^7^,^8^. Vitamin K antagonists, on the other hand, are limited by their interactions with drug and food intake leading to either an increase or decrease in anticoagulation activity. Also their activity can be nullified by food supplements containing vitamin K^8^,^9^. Thus these classes of anticoagulants require intensive coagulation monitoring. These limitations have inspired the development of novel anticoagulants that target specific enzymes or steps in the coagulation pathway. Several novel oral anticoagulants (NOACs) have been developed as alternatives to vitamin K antagonists and heparin. These NOACs function by targeting either factor Xa (FXa) (e.g. rivaroxaban and apixaban) or thrombin (e.g. dabigatran) and offer various advantages over conventional anticoagulants such as rapid onset and offset of action, predictable pharmacokinetic profile, reduced bleeding risks, non-requirement of regular laboratory monitoring, dose adjustments or dietary restrictions and fewer drug interactions. However, these medications may require dose adjustments based on patient’s renal function^10^. Treatment with NOACs is usually associated with risk of bleeding, specifically in cases of life threatening bleeding events, drug overdose or emergency surgery. The readily available antidotes to reverse their anticoagulant effect has been helpful. Specific reversal can be achieved through idarucizumab that can bind to both free and thrombin-bound dabigatran or andexanet alfa that can neutralize both direct and indirect FXa inhibitors^10^,^11^.

It has been documented that the extrinsic pathway is involved in the initiation, while the intrinsic pathway helps in the propagation of blood coagulation^12^. Thus attempts are being made to develop therapeutic strategies to block the clot initiation by inhibiting various stages in the extrinsic pathway. Among them, the ETC comprising of factor VIIa (FVIIa) and membrane-bound tissue factor (TF) play a crucial role in the clot initiation. The inhibition of this complex can control the thrombin burst and hence targeted for anticoagulant therapy^13^.

Over the years, a number of inhibitors targeting ETC have been characterized. Physiologically, tissue factor pathway inhibitor (TFPI) regulates the activity of this complex. This endogenous inhibitor has three Kunitz domains. At first, second Kunitz domain binds to FXa and subsequently, first Kunitz domain binds to FVIIa/TF forming a quaternary complex^14^. These interactions are mediated through the active sites of both serine proteases. Exogenous inhibitors like ixolaris isolated from tick salivary glands have two Kunitz domains. They form quaternary complex similar to TFPI. Interestingly, the second Kunitz domain of ixolaris binds to the exosite of FX/FXa (unlike TFPI, which binds to the active site) while the first domain binds to FVIIa/TF active site^15^. Ascaris-type inhibitors like NAPc2, although structurally distinct, exhibit a similar anticoagulant mechanism as ixolaris; they bind to FX/FXa exosite and FVIIa/TF active site^16^. Further, monoclonal antibodies and short-peptides (5–20 residues) have also been developed as inhibitors of the ETC. They bind to FX^17^ or FVIIa^18^ and block the complex formation with TF.

Snake venoms provide an alternative source of anticoagulants that specifically target the ETC^19^. They belong to phospholipase A_2_ (PLA_2_) and three-finger toxin (3FTx) families^20^. The weakly anticoagulant PLA_2_s, CM-I and CM-II, exert their activity mostly through enzymatic mechanisms, whereas the strongly anticoagulant, CM-IV inhibits the ETC both by enzymatic and non-enzymatic mechanisms^21^. We characterized a novel anticoagulant protein complex, hemextin from *Hemachatus haemachatus* venom. This tetrameric complex has two subunits, which belong to 3FTx family, that are held together by non-covalent interactions. This complex inhibits FVIIa and FVIIa/TF complex non-competitively with a Ki of 25 nM^22^. Here we report the purification and characterization of another novel anticoagulant 3FTx from the same venom that specifically and potently inhibited the activation of FX, but not of factor IX (FIX), by the ETC. However, it did not significantly inhibit the amidolytic activity of FVIIa, FVIIa/sTF complex or FXa. Thus, this specific inhibitor was named as exactin (**E**xtrinsic tenase-mediated F**X act**ivation **in**hibitor). Exactin is the first anticoagulant 3FTx that shows high identity to short-chain neurotoxins.

## Results and Discussion

Anticoagulant therapies prevent unwanted clot formation leading to death and debilitation in cardiovascular and cerebrovascular diseases. Owing to limitations of conventional anticoagulants, new classes of anticoagulants (for example, NOACs) that target individual clotting enzymes were developed. Thus, it appears that targeting specific steps in the coagulation cascade is clinically more reliable and advantageous. As described above, the extrinsic tenase complex is crucial for the initiation of the blood coagulation^23^ and hence is an important target for anticoagulation therapies. Over the years, several specific exogenous inhibitors to this complex have been characterized. Most of these inhibitors are from two key sources-saliva/salivary gland extracts of hematophagous animals and snake venoms^20^,^24^,^25^. These anticoagulants have high selectivity and distinct mechanisms of action. Such anticoagulants may provide either a potential prototype for anticoagulant therapies or help us to identify and understand susceptible interactions within the coagulation cascade and aid in developing better therapies. Among non-enzymatic anticoagulants from snake venom^20^,^24^, only hemextin targets the ETC through its direct inhibition of FVIIa^22^. Here, we describe the characterization of a novel anticoagulant 3FTx exactin from the same snake venom that inhibits FX activation by ETC by binding to both FX and FVIIa with nanomolar affinity.

### Purification of the anticoagulant protein exactin

**Figure 1:**
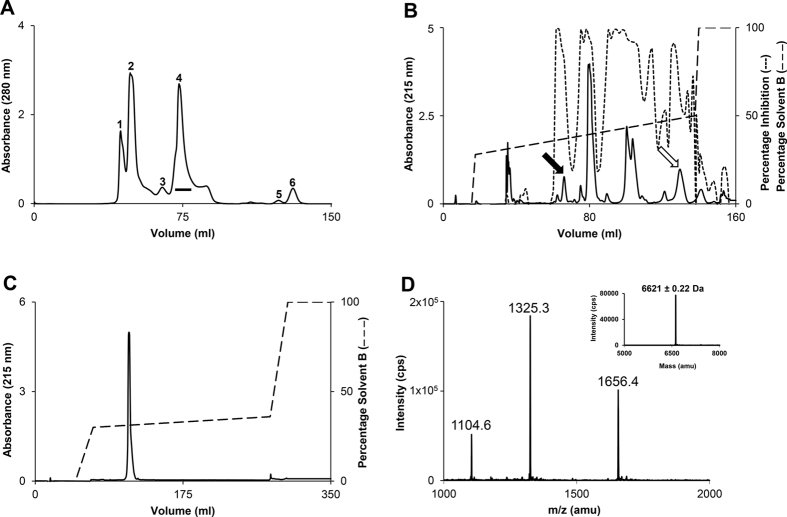
Purification of exactin from *H. haemachatus* venom. (**A**) Size-exclusion chromatography of the crude venom. The elution was monitored at 280 nm. The fractions of peak 4 (*horizontal bar*) were pooled and sub-fractionated on RP-HPLC. (**B**) RP-HPLC chromatography of peak 4. The elution was monitored at 215 nm. The inhibitory activities of the individual fractions on FX activation by the ETC were measured (*dotted line*). The peaks indicated by *solid arrow* (contains exactin) and *open arrow* (contains other anticoagulant proteins, which are being characterized) significantly prolonged the plasma prothrombin time (Fig. S1). (**C**) The re-purification of exactin on a shallow gradient of 30–36% solvent B. The elution was monitored at 215 nm. (**D**) The ESI-MS of exactin showing three peaks of mass/charge (m/z) ratio ranging from +4 to +6 charges. The mass of exactin was determined to be 6621.12 ± 0.22 Da (*inset*).

The crude *H. haemachatus* venom was size-fractionated by gel-filtration chromatography on a Superdex 30 column (Fig. 1A). Peaks 2 and 4 contained proteins that belong to PLA_2_ and 3FTx families, respectively^22^. PLA_2_s mediate their anticoagulant activity through both enzymatic and non-enzymatic mechanisms^26^,^27^. As we were interested in isolating the non-enzymatic anticoagulant proteins, we further fractionated the peak 4 on a C_18_ RP-HPLC column. The individual fractions were examined for their inhibitory activities on FX activation by the ETC (Fig. 1B)^28^. We also evaluated the effect of pooled fractions under each peak on the prothrombin time in human plasma. Although several fractions inhibited the ETC, only two pooled peaks prolonged prothrombin time significantly (>200 s at 0.2 mg/ml; Fig. S1). In the current work, we focused on the purification and characterization of anticoagulant from the peak denoted by *solid arrow*. This peak was further re-chromatographed using a shallow gradient on the same column (Fig. 1C). The homogeneity and mass of this purified anticoagulant protein was determined by ESI-MS. The protein showed 3 peaks of mass/charge (*m/z*) ratios ranging from +4 to +6 charges (Fig. 1D) and the mass of 6621.12 ± 0.22 Da (*inset*). The overall yield of purified exactin was estimated to be around 1–2 mg per 100 mg of crude venom.

### Amino acid sequence of exactin

**Figure 2:**
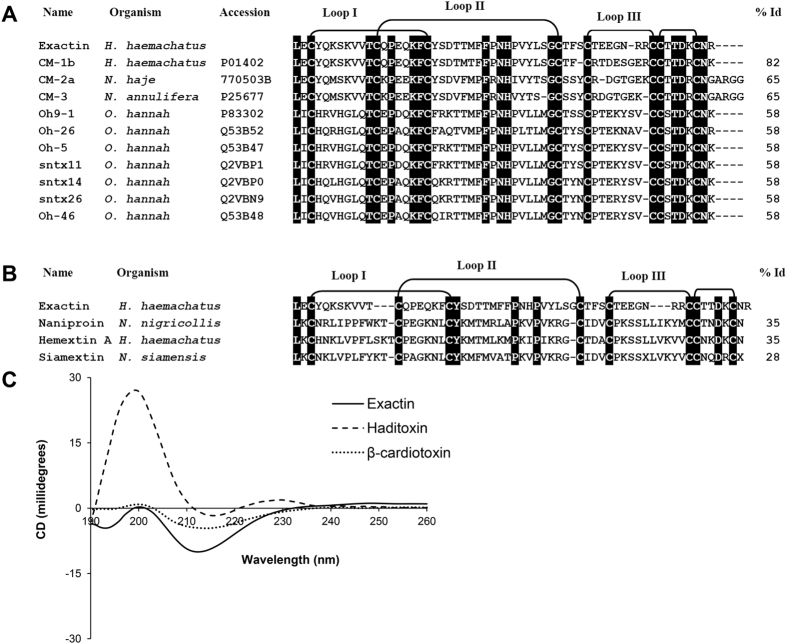
Structural characterization of exactin. (**A**) Sequence comparison with closely related 3FTxs. (**B**) Sequence comparison with anticoagulant 3FTXs. Conserved residues are highlighted in black. The sequence identity (in percentage) of each protein with exactin is shown at the end of each sequence. *H. haemachatus, Hemachatus haemachatus; N. annulifera, Naja annulifera; N. haje, Naja haje; N. nigricollis, Naja nigricollis; N. siamensis, Naja siamensis; O. hannah, Ophiophagus hannah*. (**C**) Far-UV CD spectrum of exactin showing an intense minima at 212 nm and 194 nm and maximum at 200 nm typical of β–sheeted structure. The CD spectra was compared to β-cardiotoxin and haditoxin both isolated from *O. hannah* venom.

The amino acid sequence of exactin was determined by automated Edman degradation. We were able to unequivocally identify all the residues except for the cysteines (blank cycles corresponded to conserved cysteine residues in 3FTxs). The number of cysteine residues was determined by reduction and pyridylethylation. The observed mass of 7470.91 ± 1.6 Da for the reduced and pyridylethylated protein indicates the presence of eight cysteine residues (Fig. S2). Cysteine residues in the amino acid sequence were identified by similarity with other 3FTxs and the absence of any new phenylthiohydantoin derivatives of amino acid residue in these cycles during Edman degradation. Full length protein has 57 residues and eight cysteine residues forming four disulfide bonds. The calculated mass of 6621.5 Da from the sequence matches with the observed molecular mass of 6621.12 ± 0.22 Da. BLAST search showed 82% identity to weak toxin CM1b isolated from *H. haemachatus* venom^29^ (Fig. 2A). It also showed 58% identity to a number of *O. hannah* (king cobra) neurotoxins (Fig. 2A) especially to the well characterized Ω-neurotoxin Oh9-1^30^. Interestingly, exactin showed weak identity (35% or lower) with other anticoagulant 3FTxs (Fig. 2B).

### CD spectroscopy

Exactin exhibited intense minima at 212 nm and 194 nm and maximum at 200 nm indicating predominantly β–sheeted structure (Fig. 2C). The CD spectrum is comparable to that of β–cardiotoxin, a β-blocker from the venom of *O. hannah*^31^. However, it differed significantly from that of haditoxin, a dimeric α–neurotoxin from the same venom with a minimum at 215 nm and maximum at 198–200 nm^32^.

### Anticoagulant action of exactin

**Figure 3:**
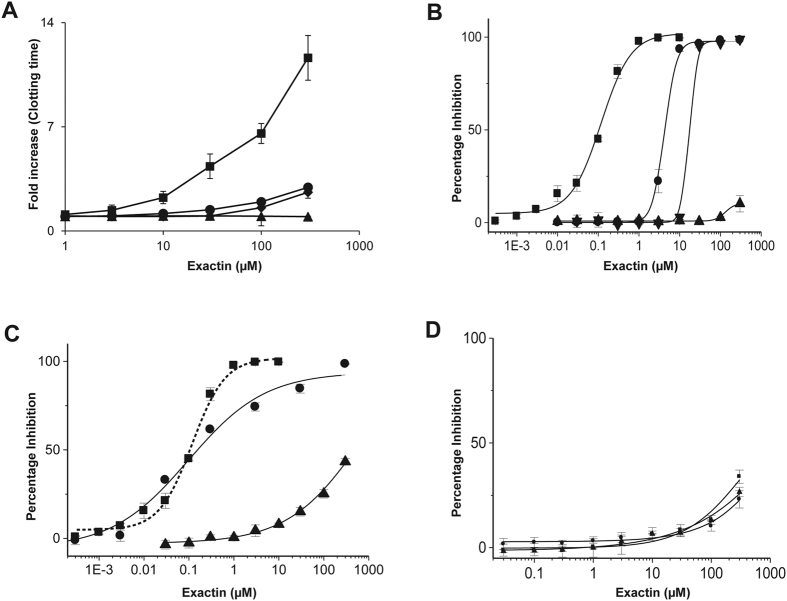
Anticoagulant activity of exactin. (**A**) Effect of exactin (1 μM to 300 μM) on the coagulation of human plasma: prothrombin time (■), Stypven time (♦), APTT (●) and thrombin time (▲). (**B**) Identification of specific site of action of exactin. Effect of exactin on reconstituted ETC (■), intrinsic tenase complex (●), prothrombinase complex (▼) and thrombin (▲). (**C**) Mechanism of inhibition of the extrinsic tenase complex. The effect of exactin on the sequential removal of each component of the ETC: removal of TF [FVIIa/FX/phospholipids (●)] and phospholipids [FVIIa/sTF/FX (▲)] compared to full complex [FVIIa/TF/FX/phospholipids (■)]. For comparison, dose-response curve of the effect of exactin on ETC is shown in dotted line. (**D**) Effect of exactin on hydrolysis of small peptides. The effect of exactin on amidolytic activity of FVIIa (●), FVIIa/sTF (■), and FVIIa/phospholipids (▲). Each data point represents the average ± SD of three independent experiments.

We determined the effect of exactin on various clotting times using a ‘Dissection Approach’^33^,^34^. Exactin significantly prolonged prothrombin time compared to Stypven time and activated partial thromboplastin time (APTT) (Fig. 3A). However, it did not have any effect on thrombin time. The results suggest that exactin specifically targets the ETC, but not the steps below activation of FX. To validate this hypothesis, we determined the effects of exactin on various reconstituted blood coagulation complexes: (a) extrinsic tenase-an assembled complex of human FVIIa with its cofactor TF in the presence of phospholipids and Ca^2+^ ions that activates FX; (b) intrinsic tenase-an assembled complex of human factor IXa (FIXa) with its cofactor factor VIIIa (FVIIIa) in the presence of phospholipids and Ca^2+^ ions that also activates FX; and (c) prothrombinase-an assembled complex of human FXa with its cofactor factor Va (FVa) in the presence of phospholipids and Ca^2+^ ions that activates prothrombin. We also studied the effects of exactin on thrombin (Fig. 3B). Exactin is a potent inhibitor of the ETC (IC_50_ = 116.49 ± 3.28 nM). It weakly inhibited FX activation by intrinsic tenase complex (IC_50_ = 4.05 ± 0.32 μM) and prothrombin activation by prothrombinase complex (IC_50_ = 17.66 ± 0.58 μM). These results suggest that exactin is a specific inhibitor of the ETC compared to the intrinsic tenase complex (>30-fold) and the prothrombinase complex (>100-fold). However, exactin failed to inhibit amidolytic activity (ability to cleave small peptidyl substrates) of thrombin (IC_50_ ≫300 μM). To further determine the protease selectivity, the effect of exactin on various coagulation and fibrinolytic proteases, such as procoagulant serine proteases: FVIIa, FXa, FIXa, factor XIa (FXIa), factor XIIa (FXIIa), α-thrombin, kallikrein, anticoagulant serine protease: activated protein C (APC) and fibrinolytic serine proteases: plasmin, urokinase and tissue plasminogen activator (t-PA) were evaluated. Exactin exhibited insignificant inhibition to their amidolytic activities with IC_50_ values ≫300 μM (Fig. S3).

### Mechanism of inhibition of the extrinsic tenase complex

To understand the molecular mechanism, the effect of exactin on ETC was examined using various assays in which each component of the ETC was removed sequentially to evaluate the role of individual components in the susceptibility of ETC. In the first experiment, cofactor TF was removed and the effect of exactin on the activation of FX by FVIIa in the presence of phospholipids was studied. The removal of TF did not affect its inhibitory potency (IC_50_ value 102.70 ± 11.71 nM compared to 116.49 ± 3.28 nM for the complete complex) (Fig. 3C). In the second experiment, we examined the effect of exactin on the FX activation by FVIIa-soluble tissue factor (sTF) complex (in the absence of phospholipids). The inhibitory potency was drastically reduced (>1000-fold) (Fig. 3C). Thus removal of phospholipids significantly altered the activity of exactin. In contrast, exactin slightly enhances the activation of FX by FVIIa in the absence of both TF and phospholipids (Fig. S4). Further, exactin is a poor inhibitor of FVIIa (FVIIa/sTF, FVIIa/phospholipids and FVIIa) amidolytic activity with IC_50_ values ≫300 μM (Fig. 3D). Thus, exactin preferably inhibits the cleavage and activation of macromolecular substrate by FVIIa/TF/phospholipids and FVIIa/phospholipid complexes.

**Figure 4:**
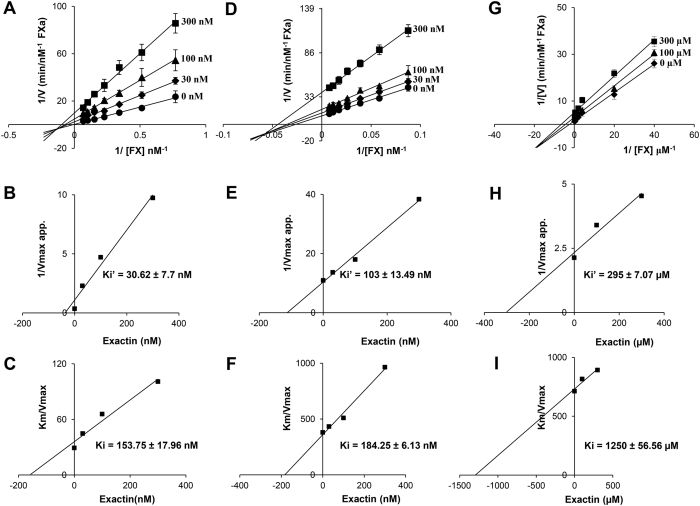
Kinetics of inhibition of extrinsic tenase complex. (**A**) The Lineweaver-Burk plot for inhibition of ETC by exactin. Both kcat and Km decreased with the increase in inhibitor concentration (Table S1), characteristic of mixed-type inhibition. (**B**,**C**) Corresponding secondary plots depicting Ki’ obtained towards the [ES] complex (FVIIa/TF/FX/phospholipids) and Ki obtained towards the [E] complex (FVIIa/TF/phospholipids). (**D**) The Lineweaver-Burk plot for the inhibition of FVIIa/phospholipids by exactin. Both kcat and Km decreased with increase in inhibitor concentration (Table S2), characteristic of mixed-type inhibition. (**E**,**F**) Corresponding secondary plots depicting Ki’ obtained towards the [ES] complex (FVIIa/FX/phospholipids) and Ki obtained towards the [E] complex (FVIIa/phospholipids). (**G**) The Lineweaver-Burk plot for inhibition of FVIIa/sTF by exactin. Both kcat and Km decreased with increase in inhibitor concentration (Table S3), characteristic of mixed-type inhibition. (**H**,**I**) Corresponding secondary plots depicting Ki’ obtained towards the [ES] complex (FVIIa/sTF/FX) and Ki obtained towards the [E] complex (FVIIa/sTF). Each data point represents the average ± SD of three to five independent experiments.

To further understand the interactions, we examined the inhibitory kinetics of exactin. It exhibited mixed-type inhibition to FX activation by the ETC (Fig. 4A) with the kinetic constants, Ki’ (towards FVIIa/TF/FX/phospholipids [ES] complex) and Ki (towards FVIIa/TF/phospholipids [E] complex) determined as 30.62 ± 7.73 nM and 153.75 ± 17.96 nM, respectively (Fig. 4B,C). Thus the affinity of the inhibitor towards [ES] was ~5-fold higher compared to [E] suggesting its preference to [ES]. The removal of TF did not alter its inhibitory mechanism (Fig. 4D). However, Ki’ for [ES] complex (FVIIa/FX/phospholipids) dropped ~3-fold to 103 ± 13.49 nM with a slight decrease in Ki of 184.25 ± 6.13 nM for the [E] complex (FVIIa/phospholipids) (Fig. 4E,F). Thus, exactin appears to bind to the complete ETC better than FVIIa/FX/phospholipids complex. FX interacts with both TF and FVIIa on the membrane surface for efficient catalysis and the exosite interactions determine the affinity of FX to the ETC^35^. The macromolecular binding exosite on FVIIa is modulated by allosteric binding of TF^36^,^37^. The unaltered affinity to [E] suggests that exactin interacts well with FVIIa even in the absence of TF. Exactin weakly inhibited FX activation in the absence of phospholipids (Fig. 4G) with the kinetic constants Ki’ and Ki of 295 ± 7.07 μM and 1250 ± 56.56 μM, respectively (Fig. 4H,I). Thus, the affinity towards the [ES] complex (FVIIa/sTF/FX) and [E] complex (FVIIa/sTF) decreased by >1000-fold, suggesting its preference towards membrane-bound complex. Phospholipids play key role in proper anchoring of FVIIa and FX on to the membrane surface via Gla domains and FX catalysis by ETC^38^,^39^,^40^. FRET analysis has shown that the active site of both FVIIa and FXa are orientated perpendicular above the membrane surface^41^,^42^. It has been hypothesized that conformational alterations of FX upon phospholipid binding enhance the susceptibility of the Arg152-Ile153 bond, presumably by influencing recognition and peptide bond hydrolysis^43^. The altered conformations in the presence of phospholipids appear to be also important for the interaction of exactin with ETC. Exactin shows low identity to cytotoxins/cardiotoxins, which are membrane-active (Fig. 2B). As expected, exactin was not membrane active as indicated by its inability to induce haemolysis of red blood cells (data not shown). Thus, increased affinity of exactin to [ES] complex with phospholipids appears to be due to altered conformation of FVIIa and FX rather than exactin’s interaction with phospholipids. The Km for FX decreases in the presence of exactin (Table S1). Thus, the binding of exactin increases the affinity of FX towards the ETC, but reduces the activation rate. As FX activation by FVIIa in the absence of TF and phospholipids was too low, the effect of exactin on FX activation by FVIIa alone was not studied.

The Km and kcat for FX activation by ETC, FVIIa/phospholipids and FVIIa/sTF in the absence of exactin (Tables S1, S2 and S3) are similar to those reported previously^38^,^44^. The lower Km for FX activation by ETC (14 nM) could be attributed to TF (Innovin) used in our assays. Innovin contains PE in addition to PC and PS, and PE enhances proteolytic activity of FVIIa/TF^45^.

### Exactin selectively inhibits the activation of factor X

**Figure 5:**
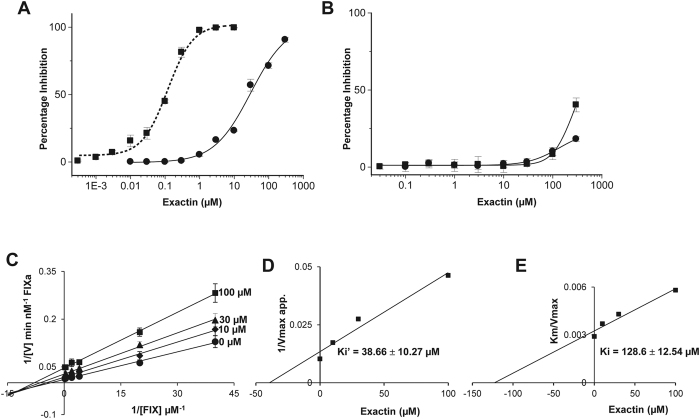
Selective inhibition of FX activation by exactin. (**A**) Effect of exactin on the activation of macromolecular substrates FX (■) and FIX (●) by ETC. For comparison, dose-response curve of the effect of exactin on ETC is shown in dotted line. (**B**) Effect of exactin on amidolytic activity of FXa (■) and FIXa (●). (**C**) Lineweaver-Burk plot for FIX activation by ETC in the presence of exactin. Both kcat and Km decreased with increase in inhibitor concentration (Table S4), characteristic of mixed-type inhibition. (**D**,**E**) Secondary plots depicting Ki’ obtained towards the [ES] complex (FVIIa/TF/FIX/phospholipids) and Ki obtained towards the [E] complex (FVIIa/TF/phospholipids). Each data point represents the average ± SD of three independent experiments.

Both FX and FIX are proteolytically activated by the ETC^46^, although the mechanisms of activation are different^47^. FX appears to be a better macromolecular substrate when compared to FIX; it has a higher affinity to phospholipid membranes (Kd for FX, 0.25 μM and Kd for FIX, 2 μM)^39^ and its rate of activation is faster compared to FIX^48^. As discussed above, exactin preferentially binds to [ES] than [E], the substrate FX appears to enhance the affinity. It was interesting to determine its ability to inhibit FIX [ES] complex. Indeed, exactin inhibited FIX activation in a dose-dependent manner with an IC_50_ value of 29.66 ± 5.27 μM (Fig. 5A). This was >100-fold higher when compared to FX activation. As with FXa, exactin is a weak inhibitor of FIXa amidolytic activity (IC_50 _≫ 300 μM) (Fig. 5B). Kinetic studies revealed exactin to be a mixed-type inhibitor with a >300-fold less affinity to FIX activation (Fig. 5C). The Ki’ and Ki values were determined as 38.66 ± 10.27 μM (Fig. 5D) and 128.6 ± 12.54 μM (Fig. 5E), respectively. The kinetic parameters Km and kcat determined for FIX activation by ETC in the absence of exactin were similar to those reported previously^49^ (Table S4). Thus exactin is a highly specific inhibitor for FX activation by the ETC.

### Exactin preferentially inhibits FX activation by extrinsic tenase complex

**Figure 6:**
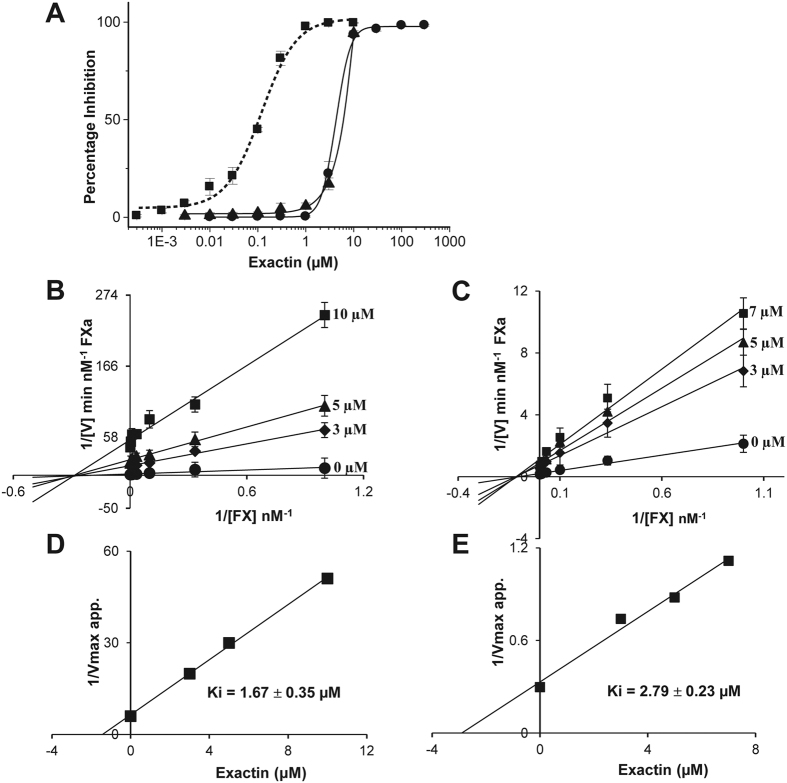
Exactin preferentially inhibits FX activation by extrinsic tenase complex. (**A**) Effect of exactin on FX activation by endogenous and exogenous FX activators. ETC (■); intrinsic tenase complex (●) and RVV-X (▲). For comparison, dose-response curve of the effect of exactin on ETC is shown in dotted line. (**B**,**C**) Lineweaver-Burk plot for FX activation by intrinsic tenase complex and RVV-X. Km remains unchanged while kcat decreased with increase in inhibitor concentration, characteristic of non-competitive inhibition (Tables S5 and S6). (**D**,**E**) The corresponding secondary plot depicting Ki. Each data point represents the average ± SD of three independent experiments.

FX is physiologically activated by two endogenous activators-extrinsic and intrinsic tenase complexes, and one exogenous activator-the snake venom Russell’s Viper Venom factor X activator (RVV-X)^50^,^51^. In all these cases, FX activation involves a proteolytic cleavage of Arg152-Ile153 bond resulting in the release of a 52-residue activation peptide. Although the same peptide bond is cleaved, these activators differ in their molecular structure and assembly. In the extrinsic and intrinsic tenase complexes, the serine proteases (FVIIa and FIXa) are similar but the cofactors (TF and FVIIIa), respectively, are distinct in their structure and properties^52^. In contrast, RVV-X is a heterotrimeric metalloprotease consisting of two snaclec subunits and a class III metalloprotease subunit^51^. Hence the effects of exactin on FX activation by these three activators were evaluated (Fig. 6A). In contrast to the effect on ETC (IC_50_ = 116.49 ± 3.28 nM), exactin exhibited >30-fold less inhibition to FX activation by both the intrinsic tenase complex and RVV-X with IC_50_ values of 4.05 ± 0.32 μM and 6.1 ± 2.9 μM, respectively. Interestingly, exactin exhibited a non-competitive inhibition by both intrinsic tenase complex (Fig. 6B,D) and RVV-X (Fig. 6C,E) with Ki values 1.67 ± 0.35 μM and 2.79 ± 0.23 μM, respectively. The kinetic parameters, Km and kcat determined for FX activation by intrinsic tenase complex and RVV-X in the absence of exactin were similar to those reported previously (Tables S5 and S6)^53^,^54^. The results further confirm the specificity of exactin towards the ETC and hence the name (**E**xtrinsic tenase-mediated F**X act**ivation **in**hibitor).

### Mechanism of anticoagulant effects of exactin and other ETC inhibitors

**Figure 7:**
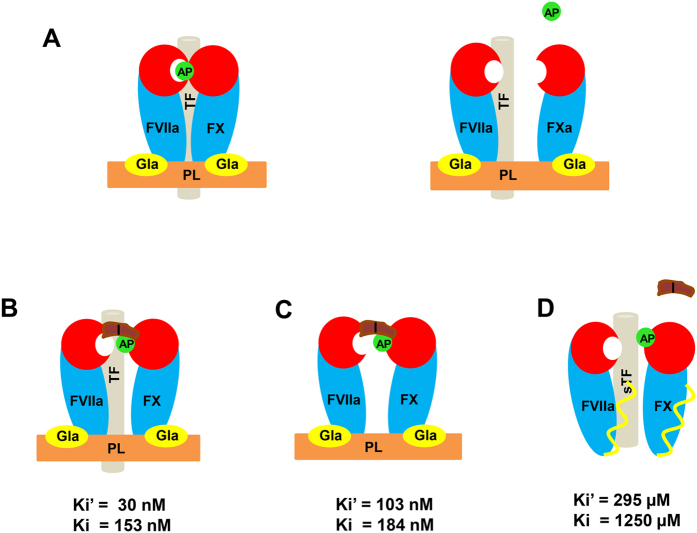
Proposed inhibitory mechanism of exactin. (**A**) Proteolytic activation of the macromolecular substrate FX by ETC via the cleavage of activation peptide (represented as AP) at Arg152-Ile153 bond. (**B**) Exactin (represented as I) exhibits a mixed-type inhibition to complete extrinsic complex by binding to a site away from the active site of FVIIa there by significantly reducing FX proteolysis. (**C**) The removal of TF from the extrinsic complex however, does not alter the binding affinity of exactin and the inhibitor is able to inhibit FX proteolysis significantly. (**D**) The removal of phospholipids (PL) from the ETC drastically reduced (>1000-folds) the inhibition of FX proteolysis by exactin. In the absence of phospholipids, the position and conformation of Gla domain may be different and thus leading to loss of affinity.

Exactin specifically inhibits FX activation by the ETC. It is a mixed-type inhibitor which prefers to bind to FVIIa-FX complex on the phospholipid membrane surface (Fig. 7) and the removal of phospholipid drastically reduced the inhibition. The affinity of exactin towards enzyme remains unaltered in the absence of TF (Fig. 4F). It did not inhibit the amidolytic activities of FVIIa or FXa (IC_50_ ≫300 μM). Thus, exactin prefers to bind to the entire extrinsic complex, but not to the active site of FVIIa or FXa, to exhibit its inhibition (Fig. 7). Thus, exactin exhibits a diverse mechanism of inhibition of ETC with nanomolar affinity. Further, it belongs to 3FTx family of toxins and thus, structurally different from the Kunitz-type and Ascaris-type inhibitors of ETC (described below). Mechanistically, exactin also differs from hemextin, a synergistically acting 3FTx complex isolated from the same venom. In contrast to exactin, hemextin inhibits the amidolytic activity of FVIIa even in the absence of TF and phospholipids with nanomolar affinity without binding to FX^22^.

To date, only a few natural anticoagulants have been characterized that bind to the extrinsic complex (FVIIa/TF/FX/phospholipids) with picomolar affinity. The Kunitz-type inhibitors, TFPI and ixolaris are FX(a)-dependent ETC inhibitors that bind to form a quaternary inhibitory complex^14^,^15^. Interestingly, TFPI binds to FXa active site, while ixolaris binds to the proheparin/heparin binding exosite on FX(a) before binding to the ETC^55^. Because of these differences in the interaction sites, TFPI inhibits FXa amidolytic activity, whereas ixolaris enhances its amidolytic activity^15^. Both TFPI and ixolaris inhibits FVIIa/TF amidolytic activity at micromolar concentrations. NAPc2, in contrast, is an Ascaris-type inhibitor that is structurally different from TFPI and ixolaris. Despite the distinct scaffold, NAPc2 exhibits scaffold FX(a)-dependent inhibition of the ETC, similar to Kunitz-type inhibitors, TFPI and ixolaris^16^. However, binding of NAPc2 to FX(a) is mediated through a novel exosite adjacent to, and partially overlapping with, the heparin binding exosite^56^. Similar to ixolaris, binding of NAPc2 to FX(a) exosite is the prerequisite for inhibition of FVIIa/TF complex. In contrast to ixolaris and similar to TFPI, NAPc2 partially inhibits FXa amidolytic activity. It also inhibits FVIIa/TF amidolytic activity at micromolar affinity. Compared to the above ETC inhibitors, NOACs target the active sites of FXa and thrombin and inhibit them with picomolar to nanomolar affinity^57^.

To summarize, we have characterized exactin, a novel 3FTx with a potent and specific anticoagulant effect on the FX activation by the ETC. Unlike other 3FTx anticoagulants which show structural similarity to cytotoxins/cardiotoxins, exactin is structurally similar to postsynaptic neurotoxins. Further studies on structure-function relationships of exactin may help in clarifying the molecular details of its interaction with ETC as well as in deciphering critical molecular surfaces of ETC that are susceptible for inhibitory control and thus, contribute to a new class of anticoagulants targeting the ETC.

## Methods

### Materials

Lyophilized *H. haemachatus* crude venom was purchased from South African Venom Suppliers (Louis Trichardt, South Africa). Reagents for thromboplastin time, thrombin time and activated partial thromboplastin time (APTT) were from Helena Laboratories (Beaumont, Texas, USA). Reagents for N-terminal sequencing were from Applied Biosystems (Carlsbad, California, USA). The chromogenic substrates, S-2222, S-2288, S-2238, S-2251, S-2444, S-2366 and S-2302 were from Chromogenix (Milano, Italy). Spectrozyme FIXa was from American Diagnostica Inc (Stamford, Connecticut, USA). Superdex 30 HiLoad (16/60) column and Jupiter C_18_ (5 μ, 300 Å, 4.6 × 250 mm) were purchased from GE Healthcare (Uppsala, Sweden) and Phenomenex (Torrance, California, USA), respectively. All other chemicals and reagents used were of the highest purity.

### Purified blood coagulation proteins and reagents

The blood coagulation factors human FVIIa, FX, FXa, FVa, FIXa, prothrombin, α-thrombin, FXIa, APC, plasmin and RVV-X were from Haemtech (Essex Junction, Vermont, USA). Factor VIII (FVIII) was purchased from American Diagnostica Inc (Stamford, Connecticut, USA). FXIIa, t-PA, urokinase and kallikrein were from Merck Chemicals Ltd. (Nottingham, UK). Recombinant human TF (Innovin) was purchased from Dade Behring (Marburg, Germany). Recombinant human soluble TF (sTF_1–218_) was a gift from Dr. Toshiyuki Miyata (National Cardiovascular Center, Suita, Japan).

### Purification of the anticoagulant protein exactin

*H. haemachatus* crude venom (100 mg in 1 ml of distilled water) was size-fractionated by gel-filtration chromatography using a Superdex 30 column equilibrated with 50 mM Tris-HCl buffer (pH 7.4) and eluted with the same buffer using an ÄKTA purifier system (GE Healthcare, Uppsala, Sweden). Peak 4 containing 3FTxs was sub-fractionated by reverse phase-high performance liquid chromatography (RP-HPLC) on a Jupiter C_18_ column (10 × 250 mm; Torrance, California, USA) equilibrated with 0.1% trifluoroacetic acid (TFA). The bound proteins were eluted using a linear gradient of 28–50% solvent B (80% acetonitrile in 0.1% TFA). The elution was monitored at 215 nm. The individual fractions were collected, lyophilized and reconstituted in calcium buffer (50 mM HEPES, pH 7.4, 140 mM NaCl, 5 mM CaCl_2_, 1% BSA). The inhibitory effects of these fractions were examined on FX activation by the reconstituted ETC (described below). Also, the fractions under each peak in RP-HPLC chromatogram were pooled and the effect of individual peaks on the prolongation of prothrombin time in human plasma was examined using a BBL fibrometer (Becton Dickinson and Co., Sparks, MD, USA). The peak indicated by the arrow was re-chromatographed using a shallow gradient on the same column. The protein fractions were injected into an API-300 LC/MS/MS (PerkinElmer Life Sciences Sciex, Waltham, Massachusetts, USA) to determine the mass and homogeneity as described previously^31^. Analyst software 1.4.1 was used to analyze and deconvolute the raw mass data.

### N-terminal sequencing

N-terminal sequencing of the native exactin was performed by automated Edman degradation using a Procise 494 pulsed liquid-phase protein sequencer (Applied Biosystems) with an on-line 785A phenylthiohydantoin derivative analyzer. The phenylthiohydantoin amino acids were sequentially identified by mapping the respective separation profiles with the standard chromatogram. The number of cysteine residues in the native protein was determined by reduction and pyridylethylation of the native exactin and examining its modified mass in electrospray ionization mass spectrometry (ESI-MS) as described earlier^58^.

### CD spectroscopy

Far-UV CD spectra (260–190 nm) were recorded using a Jasco J-810 spectropolarimeter (Jasco Corp., Tokyo, Japan) as described previously^31^. Exactin (0.5 mg/ml) was dissolved in 5 mM phosphate buffer and the measurements were carried out at room temperature using a 0.1 cm path length stoppered cuvette.

### Effect of exactin on plasma clotting times

To identify the specific stages of coagulation cascade that are inhibited, the effects of exactin on the four clotting times were examined. Citrated human blood was obtained from healthy volunteers through Tissue Repository (National University Hospital, Singapore) following the protocol approved by Institutional Review Board (NUS-IRB reference code: 08-322E) and a written informed consent from all the human subjects. Fresh plasma was obtained by centrifugation at 2600 g, 4 °C for 15 min and used for the following clotting times. The effects of various concentrations of exactin (1 μM to 300 μM) in 50 mM Tris-HCl buffer, pH 7.4 were studied on prothrombin time^59^, Stypven time^60^, thrombin time^61^ and APTT of human plasma^62^ (described below). The fibrin clot formation was monitored using a microplate coagulation test method^63^. All the experiments were done at 37 °C and the fibrin clot formation was monitored using a 96-well microplate reader (Tecan Sunrise, Männedorf, Switzerland) for 10 min at 650 nm.

### Prothrombin time

Briefly, 100 μl of plasma, 25 μl of 50 mM Tris- HCl, pH 7.4 and 50 μl of exactin were incubated for 5 min. The clotting was initiated by the addition of 25 μl of pre-warmed thromboplastin with calcium reagent and the fibrin clot formation was monitored.

### Stypven time

In a reaction well, 50 μl of plasma was incubated with 50 μl of exactin for 3 min. Pre-warmed RVV-X (50 μl, 10 ng/ml) was added and incubated for another 2 min. The clotting was initiated by the addition of 50 μl of 25 mM pre-warmed CaCl_2_ and the fibrin clot formation was monitored.

### Thrombin time

Equal volumes (50 μl) of plasma, 50 mM Tris buffer, pH 7.4 and exactin were incubated together for 5 min followed by the addition of 50 μl pre-warmed thrombin time reagent (0.15 NIH units) and the fibrin formation was monitored.

### APTT

Equal volumes (50 μl) of plasma and exactin were incubated for 3 min. Pre-warmed APTT reagent (50 μl) was added and further incubated for 2 min after which the clotting was initiated by the addition of 50 μl of pre-warmed 25 mM CaCl_2_ and the fibrin formation monitored.

### Effect of exactin on FX activation by ETC

In a reaction volume of 200 μl, the ETC was reconstituted by incubating FVIIa (10 pM) with recombinant human TF (Innovin) in calcium buffer for 15 min at 37 °C. After 15 min incubation with various concentrations of exactin (25 μl; 300 pM to 10 μM), FX (25 μl) was added to make a final concentration of 30 nM. The activation was quenched after 15 min by adding 50 μl of EDTA buffer (50 mM HEPES pH 7.4, 140 mM NaCl, 50 mM EDTA, 1% BSA). The initial reaction velocity of S-2222 cleavage by FXa formed in the reaction was measured by the hydrolysis of 50 μl of 500 μM S-2222 in a microplate reader at 405 nm. The amount of FXa generated was calculated using a FXa standard curve. FXa formed in the absence of inhibitor was considered as 100% and the IC_50_ value (inhibitor concentration which shows 50% activity) was determined.

### Effect of exactin on FX activation by intrinsic tenase complex

The intrinsic tenase complex was reconstituted according to the method of Zhang *et al*.^64^ with minor modifications. Briefly, all the reactions were carried out in calcium buffer having 67 μM of reconstituted phospholipids [phospholipid vesicles (phosphatidylcholine: phosphatidylserine {7:3} from Avanti Polar Lipids) was prepared in 50 mM HEPES, pH 7.4 as described earlier^65^] at 37 °C. In a reaction volume of 200 μl, FVIII (5 nM) was incubated for 10 min with thrombin (500 pM) and the reaction was quenched by the addition of 25 μl hirudin (115 units/ml/well), a thrombin inhibitor. To this FIXa (25 μl) was added to make a final concentration of 1 nM. After 10 min, varying concentrations of exactin (25 μl; 30 nM to 300 μM) was added. The macromolecular substrate, FX (25 μl) was then added to make a final concentration of 25 nM. The reaction was quenched after 15 min by adding the EDTA buffer. The hydrolysis of 25 μl of 500 μM S-2222 by the FXa formed was measured at 405 nm and IC_50_ value was determined as mentioned above. We have also determined the kinetics of inhibition in the presence of exactin (3 μM, 5 μM and 10 μM) with varying concentrations of FX (0.001 μM to 1 μM). Lineweaver-Burke plots were used to determine the type of inhibition.

### Effect of exactin on the activation of FX by RVV-X

All the reactions were carried out in calcium buffer at 37 °C. Briefly, 50 μl of exactin (300 pM to 10 μM) was incubated with 25 μl of RVV-X (100 pM) in a 200 μl reaction well for 15 min. The FX activation was initiated by the addition of 25 μl of FX (12.5 nM). The FXa formation was quenched after 15 min by the addition of 50 μl of EDTA buffer and the hydrolysis of 50 μl of 500 μM S-2222 by FXa generated was measured at 405 nm. The IC_50_ value was determined as mentioned above. We have also determined the kinetics of inhibition in the presence of exactin (3 μM, 5 μM and 7 μM) with varying concentrations of FX (0.001 μM to 1 μM). Lineweaver-Burke plots were used to determine the type of inhibition.

### Effect of exactin on the activation of prothrombin by the prothrombinase complex

The assay was carried out in calcium buffer with a phospholipid concentration of 67 μM. In individual wells of a 96-well plate, varying concentration of exactin (50 μl; 30 nM to 300 μM) was added to a reconstituted FXa (10 pM)–FVa (1 nM) complex in a total volume of 200 μl and incubated at 37 °C for 15 min. 25 μl of prothrombin (12.5 nM) was added and incubated for another 15 min. The reaction was then quenched by 50 μl of EDTA buffer. The hydrolysis of 50 μl of 250 μM S-2238 by thrombin generated in the reaction mix was measured in a multiplate reader at 405 nm. The amount of thrombin generated at each concentration of exactin was determined from a standard curve and the IC_50_ value was calculated considering thrombin generated in the absence of inhibitor as 100%.

### Mechanism of inhibition of the extrinsic tenase complex

To examine the role of phospholipids, various concentrations of exactin (50 μl; 30 nM to 300 μM) was incubated with FVIIa (10 nM) and sTF (30 nM) in calcium buffer containing 2.5 mM MgCl_2_ for 15 min before the addition of 25 μl FX (2 μM final concentration). To examine the role of TF, 25 μl of FVIIa (10 nM and 20 nM in calcium buffer with or without phospholipids [67 μM] in the absence of TF, respectively) was incubated with various concentrations of exactin (50 μl; 30 nM to 300 μM) for 15 min. FX (25 μl) was then added to make a final concentration of 640 nM. In all experiments, the reaction was quenched after 15 min of FX activation by the addition of EDTA buffer and the amount of FXa formed was measured as described above.

The kinetics of inhibition of FX activation by ETC, FVIIa/sTF and FVIIa in the presence of phospholipids was determined at various substrate concentrations. For FX activation by ETC, effect of exactin (30 nM, 100 nM, 300 nM) on FX (0.58 nM to 50 nM) activation was examined. For kinetic studies in the absence of phospholipids, the effect of exactin (100 μM, 300 μM) on FX (0.025 μM to 5 μM) activation was examined. In case of FX activation by FVIIa in the presence of phospholipids and absence of tissue factor, effect of exactin (30 nM, 100 nM, 300 nM) on FX (0.011 μM to 1 μM) activation was examined. Lineweaver-Burk plots were used to determine the type of inhibition.

### Effect of exactin on amidolytic activity of coagulation proteases

The effect of exactin (30 nM to 300 μM) on amidolytic activities of various serine proteases was evaluated: procoagulant serine proteases: FVIIa [in presence of sTF (FVIIa- 10 nM, sTF-30 nM), without sTF (FVIIa-300 nM), with phospholipids and without TF (FVIIa-300 nM)], FXa [1 nM], FIXa [300 nM], FXIa [0.125 nM], FXIIa [20 nM], α-thrombin [3 nM], kallikrein [1 nM]), anticoagulant serine protease APC [2.5 nM] and fibrinolytic serine proteases (plasmin [3.6 nM], urokinase [40 units/ml] and t-PA [37 nM]). All the experiments were done at 37 °C. The hydrolysis of chromogenic substrates S-2222 (500 μM/FXa), S-2288 (500 μM/FVIIa; 1 mM/t-PA), S-2238 (250 μM/α-thrombin), S-2251 (1.2 mM/plasmin), S-2444 (0.3 mM/urokinase), S-2366 (0.67 mM/APC; 1 mM/FXIa), S-2302 (1 mM/FXIIa, kallikrein) and Spectrozyme FIXa (1 mM) were measured at 405 nm.

### Effect of exactin on FIX activation by extrinsic tenase complex

The dose-response effect of exactin (0.01 μM to 300 μM) on FIX activation by ETC was examined at 37 °C in calcium buffer. Briefly, the ETC was reconstituted from 1 nM FVIIa and Innovin. The tenase complex was incubated with various concentrations of exactin (25 μl). FIX was then added to the reaction mixture to make a final concentration of 600 nM (25 μl). After 15 min of incubation, the reaction was quenched by adding EDTA buffer and the hydrolysis of the chromogenic substrate, 100 μl of Spectrozyme FIXa (1 mM) by FIXa generated in the reaction mixture was measured at 405 nm. From a FIXa standard curve, the amount of FIXa formed at each concentration of exactin was measured. This was used to determine the IC_50_ value, considering FIXa generated in the absence of inhibitor as 100%. We have also determined the kinetics of inhibition in the presence of exactin (10 μM, 30 μM and 100 μM) with varying concentrations of FIX (0.025 μM to 5 μM). Lineweaver-Burke plots were used to determine the type of inhibition.

### Data analysis

Data obtained from these inhibition studies were fitted to the following equations for mixed-type (Eq. 1) and non-competitive (Eq. 2) inhibition, respectively^66^.


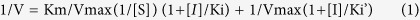



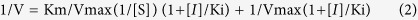


For a mixed-type inhibition where the inhibitor is capable of binding to both [E] and [ES] complex with different affinities, the data from the Lineweaver-Burk plot were re-plotted as *Km*/*Vmax* {*1* + [I]/*Ki*} (the slope) versus [I] or as *1/Vmax app.* (the y-axis intercept) versus [I], respectively. The x-axis intercept for the respective secondary plots would give Ki (affinity towards [E]) and Ki’ (affinity towards [ES] complex). In the case of non-competitive inhibition, the data from the Lineweaver-Burk plot was re-plotted as *1/Vmax app.* (the y-axis intercept) versus inhibitor concentration to determine Ki.

## Additional Information

**How to cite this article**: Girish, V. M. and Kini, R. M. Exactin: A specific inhibitor of Factor X activation by extrinsic tenase complex from the venom of *Hemachatus haemachatus. Sci. Rep.* **6**, 32036; doi: 10.1038/srep32036 (2016).

## Supplementary Material

Supplementary Information
