# Supplementary material for: Exactin: A specific inhibitor of Factor X activation by extrinsic tenase complex from the venom of Hemachatus haemachatus
Source: Sci Rep. 2016 Aug 25;6:32036. doi: 10.1038/srep32036 (PMC4997346; doi:10.1038/srep32036)
Supplement: Supplementary Information [file srep32036-s1.pdf]

Supporting Information for

Exactin: A specific inhibitor of Factor X activation by extrinsic tenase complex from the venom of *Hemachatus haemachatus*

Vallerinteavide Mavelli Girish[*] and R. Manjunatha Kini[*, §, ¶]

*Department of Biological Sciences, Faculty of Science, National University of Singapore, Singapore 119260, Singapore, §Department of Biochemistry, Medical College of Virginia, Virginia Commonwealth University, Richmond, Virginia 23298, USA, ¶University of South Australia, School of Pharmacy and Medical Sciences, Adelaide, South Australia 5001, Australia.

Supplementary figures:

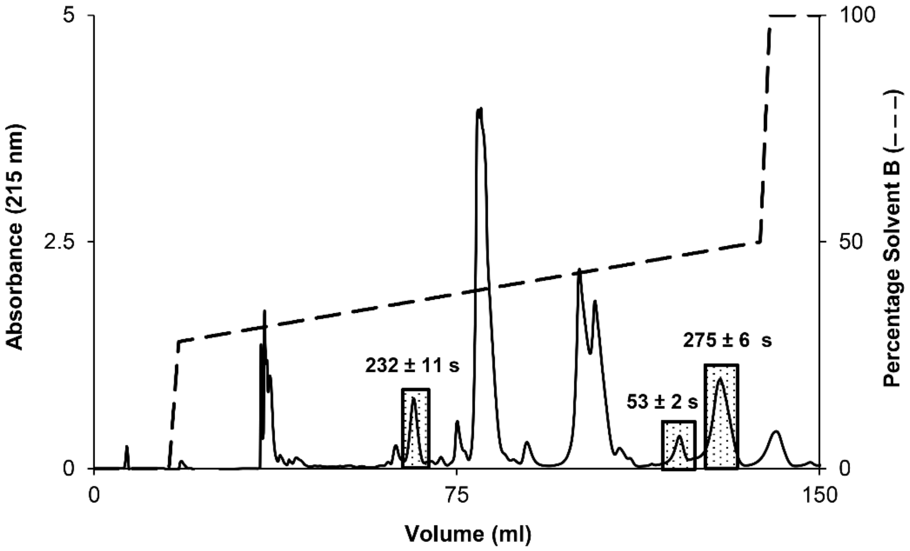

Fig. S1. Effect of pooled RP-HPLC fractions of peak 4 on prothrombin time. The fractions under each peak in RP-HPLC chromatogram (**Fig.1B**) were pooled and the effect of individual peaks on the prolongation of prothrombin time in human plasma was examined using a BBL fibrometer (Becton Dickinson and Co., Sparks, MD, USA). Only two pooled fractions (exactin peak denoted by *solid arrow* and peak denoted by *open arrow* in **Fig.1B**) prolonged the prothrombin time significantly in the plasma (>200 s at 0.2 mg/ml).

A

LEXYQKSKVVTXQPEQKFXYSDTTMFFPNHPVYLSGXTFSXTEEGNRRXXTTDKXNR

B

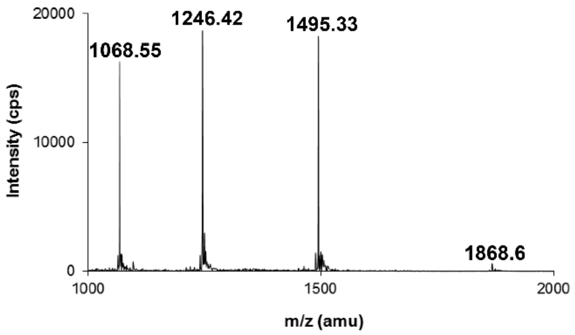

Fig.S2. Reduction and pyridylethylation of exactin. (A) The complete sequence of exactin derived by Edman degradation with residues corresponding to cysteines denoted by ‘X’ (blank cycles corresponded to conserved cysteine residues in 3FTxs). **(B)** The ESI-MS of S-pyridylethylated exactin showing the four peaks of mass/charge (m/z) ratio ranging from +4 to +7 charges. The mass determined to be 7470.91 ± 1.6 Da corresponds to eight cysteine residues in the protein.

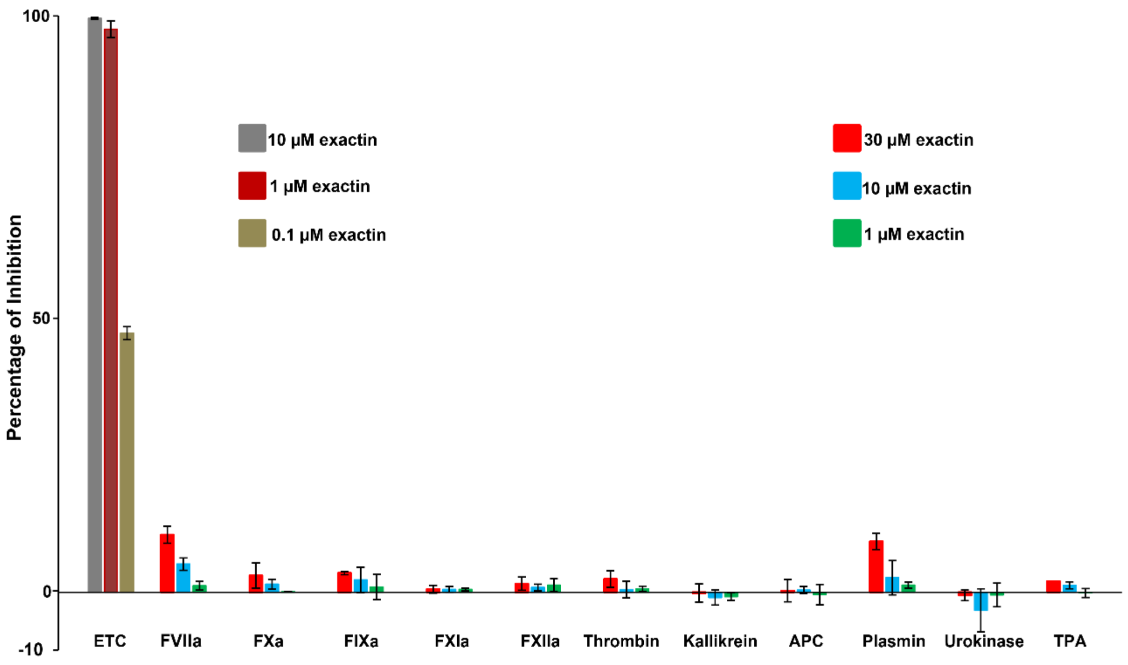

Fig.S3. Serine protease specificity of exactin. Exactin was screened against 10 serine proteases: procoagulant serine proteases (FVIIa [300 nM], FXa [1 nM], FIXa [300 nM], FXIa [0.125 nM], FXIIa [20 nM], α-thrombin [3 nM], kallikrein [1 nM]), anticoagulant serine protease APC [2.5 nM] and fibrinolytic serine proteases (plasmin [3.6 nM], urokinase [40 units/ml] and t-PA [37 nM]). Exactin is a poor inhibitor with IC_{50} values >>300 µM when compared to the inhibition to the full extrinsic tenase complex (ETC) [IC_{50} 116 ± 3.28 nM]. All the experiments were done at 37°C. The hydrolysis of chromogenic substrates S-2222 (500 µM/FXa), S-2288 (500 µM/FVIIa; 1 mM/t-PA), S-2238 (250 µM/α-thrombin), S-2251 (1.2 mM/plasmin), S-2444 (0.3 mM/urokinase), S-2366 (0.67 mM/APC; 1 mM/FXIa), S-2302 (1 mM/FXIIa, kallikrein) and Spectrozyme FIXa (1 mM) were measured at 405 nm. Each data point represents the average ± SD of three independent experiments.

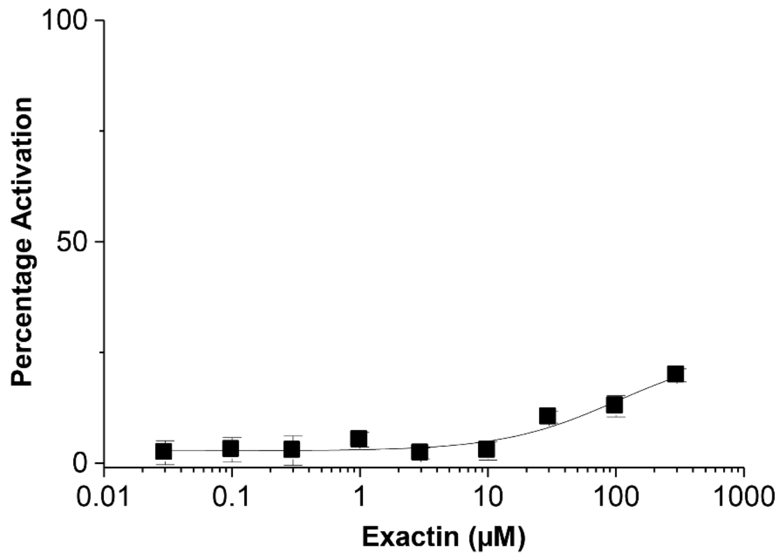

Fig.S4. Effect of exactin on FX activation by FVIIa in the absence of TF and phospholipids. A slight enhancement in FX activation was observed. Each data point represents the average of three independent experiments.

Supplementary tables:

Table S1. Kinetic parameters: Effect of exactin on FX activation by FVIIa/TF/phospholipids

Exactin (nM)	Km (nM)	Kcat (s^{-1})	Kcat/Km (s^{-1} nM^{-1})
0	14.57 ± 0.87	1.7 ± 0.017	0.117 ± 0.055
30	8.31 ± 0.72	0.95 ± 0.98	0.114 ± 0.008
100	6.46 ± 1.98	0.623 ± 0.2	0.095 ± 0.012
300	5.66 ± 1.9	0.32 ± 0.124	0.057 ± 0.013

Table S2. Kinetic parameters: Effect of exactin on FX activation by FVIIa/phospholipids

Exactin (nM)	Km (nM)	Kcat (s^{-1})	Kcat/Km (s^{-1} nM^{-1})
0	36.56 ± 2.76	0.0558 ± 0.0046	0.0015 ± 0.00014
30	23.75 ± 2.71	0.0346 ± 0.0026	0.00145 ± 0.0002
100	18.37 ± 1.49	0.0262 ± 0.0035	0.00142 ± 0.0002
300	12.22 ± 1.029	0.0151 ± 0.0031	0.0013 ± 0.00026

Table S3. Kinetic parameters: Effect of exactin on FX activation by FVIIa/sTF

Exactin (µM)	Km (µM)	Kcat (s^{-1})	Kcat/Km (s^{-1} $µM^{-1}$)
0	1.03 ± 0.089	0.0017± 6.13 E-05	0.0016 ± 0.0001
100	0.76 ± 0.126	0.0011 ± 6.01 E-06	0.0014 ± 0.0002
300	0.42 ± 0.022	0.0004 ± 6.66 E-05	0.0009 ± 0.0002

Table S4. Kinetic parameters: Effect of exactin on FIX activation by FVIIa/TF/phospholipids

Exactin (µM)	Km (nM)	Kcat (s^{-1})	Kcat/Km (s^{-1} nM^{-1})
0	299.13 ± 7.84	1.34 ± 0.012	0.0044 ± 0.00015
10	231.83 ± 6.83	0.94 ± 0.0007	0.004 ± 0.0001
30	189± 11.72	0.744 ± 0.018	0.0039 ± 0.00017
100	156.2 ± 2.74	0.353 ± 0.01	0.0022 ± 0.00006

Table S5. Kinetic parameters: Effect of exactin on FX activation by FIXa/FVIIIa/phospholipids

Exactin (µM)	Km (nM)	Kcat (s^{-1})	Kcat/Km (s^{-1} nM^{-1})
0	4.31 ± 0.62	0.48 ± 0.064	0.11 ± 0.012
3	3.26 ± 0.46	0.07 ± 0.0024	0.021 ± 0.002
5	3.21 ± 0.24	0.042 ± 0.004	0.013 ± 0.002
10	3.23 ± 0.24	0.022 ± 0.004	0.007 ± 0.002

Table S6. Kinetic parameters: Effect of exactin on FX activation by RVV-X

Exactin (µM)	Km (nM)	Kcat (s^{-1})	Kcat/Km (s^{-1} nM^{-1})
0	8.48 ± 1.06	0.85 ± 0.027	0.101 ± 0.01
3	8.49 ± 0.55	0.23 ± 0.044	0.0272 ± 0.006
5	9.19 ± 0.53	0.19 ± 0.023	0.021 ± 0.002
7	8.56 ± 0.32	0.15 ± 0.018	0.017 ± 0.0015
